# Smoking, Alcohol Consumption, and Risks for Biliary Tract Cancer and Intrahepatic Bile Duct Cancer

**DOI:** 10.2188/jea.JE20180011

**Published:** 2019-05-05

**Authors:** Takeshi Makiuchi, Tomotaka Sobue, Tetsuhisa Kitamura, Norie Sawada, Motoki Iwasaki, Taiki Yamaji, Taichi Shimazu, Manami Inoue, Shoichiro Tsugane

**Affiliations:** 1 Department of Social and Environmental Medicine, Graduate School of Medicine, Osaka University, Osaka, Japan; 2 Epidemiology and Prevention Group, Center for Public Health Sciences, National Cancer Center, Tokyo, Japan

**Keywords:** smoking, alcohol consumption, BTC, IHBDC

## Abstract

**Background:**

Smoking and alcohol are established risk factors for several types of cancer, but the effects on biliary cancers, comprising biliary tract cancer (BTC) and intrahepatic bile duct cancer (IHBDC), have been inconclusive.

**Methods:**

In this population-based prospective cohort study in Japan, we investigated the association of smoking and alcohol consumption with the risks of BTC and its subtypes and IHBDC incidence in men and women. Furthermore, the association of smoking stratified by drinking status was investigated. The hazard ratios (HRs) and 95% confidence intervals (CIs) were calculated using the Cox proportional hazard model.

**Results:**

A total of 48,367 men and 54,776 women aged 40–69 years were enrolled from 1990 through 1994 and followed up for 846,417 person-years in men and 1,021,330 person-years in women until 2012, during which 246 BTC and 80 IHBDC male cases and 227 BTC and 60 IHBDC female cases were identified. In men, smoking was significantly associated with an increased risk of IHBDC (HR 2.25; 95% CI, 1.19–4.25 for current smokers with ≥30 pack-years compared with non-smokers), and the risk was enhanced among regular drinkers compared with non/occasional-drinkers (HR 3.48; 95% CI, 1.41–8.61). A non-significant increase of IHBDC risk associated with alcohol was observed. Neither smoking nor alcohol consumption was associated with BTC risk. In women, the association of smoking and alcohol consumption with IHBDC and BTC was unclear because current smokers and regular drinkers were very few.

**Conclusion:**

Our findings suggest that smoking increases IHBDC risk in men, especially among regular drinkers.

## INTRODUCTION

Biliary cancers, including biliary tract cancer (BTC) and intrahepatic bile duct cancer (IHBDC), are highly fatal malignancies. BTC comprises gallbladder cancer (GBC), extrahepatic bile duct cancer (EHBDC), and ampulla of Vater cancer (AVC). The incidence of BTC is globally rare, but relatively higher in East Asia.^1^ Chronic inflammation is strongly suggested as one of the major risk factors of BTC,^2^^–^^4^ although its etiology is poorly understood due to its low incidence. IHBDC is a cancer that arises on a bile duct within a liver and is histologically equivalent to EHBDC. However, IHBDC is classified as primary liver cancer (PLC) and accounts for approximately 5% of PLC in Japan.^5^ Infestation with liver flukes is a main known cause of IHBDC, and IHBDC is suggested to share some risk factors with both HCC (eg, hepatitis virus infection, cirrhosis)^6^^–^^10^ and BTC (eg, primary sclerosing cholangitis).^11^^,^^12^

Smoking has been well-studied and is considered causally related to several types of cancers.^13^ Smoking may cause cancer in the epithelium of the biliary tract because carcinogenic products, such as benzopyrene, are metabolized by hepatic microsomes and excreted to bile,^14^ but the association with BTC or IHBDC risk in humans is inconclusive because the epidemiological studies to date have been inconsistent and retrospective in design.^7^^,^^13^^,^^15^^,^^16^

Alcohol has been identified as carcinogenic for several types of cancers, including liver cancer.^13^^,^^17^ The association of alcohol consumption with IHBDC risk has been inconclusive because, although several previous studies showed positive associations, all were retrospective studies.^13^ The carcinogenic effect of alcohol consumption on the biliary tract is controversial because an anti-carcinogenic effect caused by inhibiting cholesterol metabolism leading to decreased gallstone formation has been suggested.^18^^,^^19^ A null effect of alcohol consumption on BTC has been shown in many of the previous studies.^4^^,^^15^^,^^20^^–^^23^ Furthermore, alcohol consumption may modify the effect of smoking on the liver and biliary tract, but it is not clear if the direction of modification is stimulating or inhibiting because ethanol may either induce enzymes that metabolize procarcinogen to carcinogen or enzymes that are responsible for detoxification of the produced carcinogen.^24^ The interaction in humans has not been well-studied for biliary cancers.

Therefore, in the present study, we investigated the association of smoking and alcohol consumption with the risks of BTC and its subtypes and IHBDC incidence in men and women in a large-scale, population-based prospective cohort study in Japan, with special focus on the difference of the risks between these cancers. Furthermore, we investigated the effect modification of alcohol consumption on smoking.

## METHODS

### Study cohort and participants

The Japan Public Health Center-based Prospective Study (JPHC Study) is a cohort study that mainly investigates non-communicable diseases. This study comprises two cohorts, consisting of 140,420 participants from 11 public health centers (PHCs). The design of this study has been reported in detail elsewhere.^25^ The JPHC study was approved by the Institutional Review Board of the National Cancer Center, Tokyo, Japan. The present study was approved by the Ethical Review Board of Osaka University, Osaka, Japan.

Participants from one PHC area (Katsushika PHC; 7,097 participants) in Cohort I were excluded because cancer incidence data were not collected. Participants were also excluded for the following reasons: non-Japanese nationality (*n* = 51); late report of relocation out of the study area before the start of follow-up (*n* = 188); ineligibility owing to an incorrect date of birth (*n* = 7); and duplicate registration (*n* = 10). After excluding these ineligible participants, 106,324 participants responded to the baseline survey (approximately 79.9% response rate). Of the eligible responders, we excluded the participants for whom either smoking or alcohol consumption information was missing (*n* = 2,934) or date of end of follow-up was missing (*n* = 243), and the participants who were diagnosed as BTC or IHBDC before responding to the baseline survey (*n* = 4).

### Exposure assessment

As a baseline survey, a self-administered questionnaire was distributed in 1990 for Cohort I and in 1993–1994 for Cohort II. The questionnaire asked about a variety of lifestyle factors, including smoking and alcohol habits.

For smoking habits, the questionnaire asked about smoking status (never, former, or current smoker), age at initiation, age at cessation (former smoker only) and average number of cigarettes smoked per day. Participants were divided into the following four smoking groups: “never smoker”, “former smoker”, “current smoker with <30 pack-years”, and “current smoker with “≥30 pack-years”. Pack-years, used as an indicator of smoking intensity for current smokers, was calculated by multiplying the years of smoking by the number of cigarettes per day divided by 20. In the analysis for women, participants were divided in the following two groups because female smokers were very few: “never smoker” and “former/current smoker”.

For alcohol consumption, in Cohort I, the questionnaire asked about frequency (<1 day/month, 1–3 days/month, 1–2 days/week, 3–4 days/week, 5–6 day/week, or everyday), then asked about types of beverage and average volume of daily consumption to those who consume alcoholic beverage at least 1 day/week. In Cohort II, the questionnaire asked about drinking status (never, former, or current drinker), then asked about frequency (1–3 days/month, 1–2 days/week, 3–4 days/week,or almost every day), types of beverages, and average volume of daily consumption to former and current drinker. Participants were divided into the following four drinking groups: “nondrinkers (<1 day/month in Cohort I and II or former drinkers in Cohort II)”, “occasional drinkers (1–3 days/month)”, “regular drinkers (≥1 day/week) with <300 g/week ethanol consumption”, and “regular drinkers with ≥300 g/week ethanol consumption”. We followed the previous studies to calculate the amount of ethanol.^26^^,^^27^ In the analysis for women, participants were divided in the following two groups because female drinkers were very few: “non/occasional drinker” and “regular drinker”. The validity and reproducibility of alcohol consumption estimated from the questionnaire by the cohort was assessed using dietary records for 28 days. Spearman’s correlation coefficients were 0.79 in men and 0.44 in women for validity and 0.78 in men and 0.66 in women for reproducibility. Mean daily alcohol consumption from the questionnaire was comparable to the dietary record.^28^

### Follow-up and case identification

Follow-up was conducted using information about residential status and survival collected from the residential registers of each municipality in the study area. Information on the cause of death was supplemented with death certificate information, with permission from the Ministry of Health, Labor and Welfare (MHLW) in Japan. Death certificates were coded in accordance with the requirements of MHLW. Of the eligible participants, 10,909 moved out of the study area, 162 were lost to follow-up, 16 withdrew from the study, and 18,286 died during the at-risk period.

The incidence of cancer was identified mainly using two data sources: active patient notification from major local hospitals in the study area and population-based cancer registries. Death certificate information was used as a supplementary information source. The site of origin and histological cancer type were coded using the International Classification of Diseases for Oncology, 3rd Edition, with IHBDC as C22.1, GBC as C23.9, EHBDC as C24.0, AVC as C24.1, the overlapping sites of the biliary tract as C24.8, and unspecified as C24.9; in the present analysis, BTC included C23.9, C24.0, C24.1, C24.8, and C24.9. If a participant was diagnosed as having more than one cancer out of these biliary cancers, that with the earliest diagnosis date was used for the analysis. The proportion of cases where incidence was ascertained using death certificates only was 10.6% for BTC and IHBDC, and 6.2% for all types of cancer.

### Statistical analysis

The number of person-years of follow-up was calculated from the date of the baseline survey until the end of follow-up, which was the earliest date out of the following: date of moving out of the study area, loss to follow-up, withdrawal from the study, death, diagnosis of biliary cancer, or the last date of the follow-up period (December 31, 2012). In the present study, follow-up was not ended when the participants were diagnosed with a cancer other than BTC and IHBDC.

Hazard ratios (HRs), 95% confidence intervals (CIs), *P*-trend, and *P*-interaction for IHBDC and BTC and its subtypes (GBC, EHBDC, and AVC) were estimated in men and women using the Cox proportional hazards model with adjustment for potential confounders. For the analysis of alcohol consumption, we excluded participants with a history of chronic hepatitis or cirrhosis because these participants might change their drinking behavior (*n* = 1,762).^29^ This multivariate analysis model was adjusted for the following factors: age (continuous), study area (10 PHC areas), body mass index (<23, ≥23 and <25, ≥25 and <27, ≥27 kg/m^2^, or unknown), history of cholelithiasis (no or yes), history of diabetes mellitus (no or yes), history of chronic hepatitis or cirrhosis (not applicable when estimate HRs for alcohol consumption: no or yes), Japanese tea consumption (<1, 1–2, 3–4, ≥5 cup[s]/day, or unknown), smoking (not applicable when estimate HRs for smoking: never smokers, former smokers, current smokers with <30 pack-years, or current smokers with ≥30 pack-years), alcohol consumption (not applicable when estimate HRs for alcohol consumption: non-drinkers, occasional drinkers, regular drinkers with ethanol consumption of <300 g/week, and regular drinkers with ethanol consumption of ≥300 g/week). Additionally, HRs for smoking status stratified by drinking status (non/occasional drinkers vs regular drinkers) were estimated.

*P*-trend was calculated by assigning ordinal values to each category and entering as a continuous term in each model. *P*-interaction to evaluate effect modification of alcohol consumption (non/occasional drinkers vs regular drinker) on smoking was calculated by assigning ordinal values to each variable and creating interaction term by multiplying ordinal values for each variable. All *P* values reported were two-sided, and the significance level was set at *P* < 0.05. All statistical analyses were performed using Stata Version 13 (Stata Corporation, College Station, TX, USA).

## RESULTS

Baseline characteristics of the participants by smoking and alcohol consumption are shown in Table 1. A total of 103,143 participants (48,367 men and 54,776 women) were included in the analysis population and followed up for 1,867,747 person-years (846,417 in men and 1,021,330 in women). During the follow-up period, 246 cases of BTC (70 GBCs, 140 EHBDCs, 26 AVCs, 10 unspecified location, and no cases of overlapping site), and 80 cases of IHBDC were identified in men, and 227 cases of BTC (119 GBCs, 78 EHBDCs, 16 AVCs, 14 unspecified location, and no cases of overlapping site), and 60 cases of IHBDC were identified in women. The proportions of smokers and regular drinkers in women were remarkably lower than those in men. The proportion of regular drinkers was higher among smokers than that among never smokers, and the proportion of current smokers was higher among regular drinkers than that among non-/occasional drinkers in men and women.

**Table 1.** Characteristics of the study participants at baseline

	Smoking status				Alcohol consumption			
	
	Never Smoker	Former Smoker	Current Smoker (pack-year)		Non Drinker	Occasional Drinker	Regular Drinker (ethanol/week)	
	
			(<30)	(≥30)			(<300 g)	(≥300 g)
Men								
Number of subjects	11,805	11,381	11,127	14,054	10,636	4,187	20,689	11,641
Person-years	213,060.1	199,244.8	196,699.2	237,413.4	181,021.0	76,850.3	363,215.8	207,917.2
Age, years, mean (SD)	51.8 (7.6)	53.3 (8.3)	48.2 (7.4)	52.6 (7.6)	53.6 (8.5)	50.2 (7.4)	51.2 (8.0)	50.7 (7.2)
BMI, kg/m^2^, mean (SD)	23.9 (2.8)	23.8 (2.8)	23.1 (2.8)	23.2 (2.9)	23.3 (3.0)	24.0 (3.0)	23.4 (2.7)	23.5 (2.8)
History of cholelithiasis, Yes, %	3.0	3.2	2.2	2.7	3.4	3.2	2.4	2.3
History of diabetes mellitus, Yes, %	5.8	7.3	5.6	7.5	7.5	6.1	5.8	6.4
History of chronic hepatitis or cirrhosis, Yes, %	2.0	2.8	2.4	2.8	—	—	—	—
Japanese tea consumption, ≥1/day, %	70.1	75.4	70.3	77.2	73.4	69.3	74.9	72.2
Current smoker, %	—	—	—	—	46.9	46.7	50.2	61.7
Regular drinker, ≥1/week, %	61.2	68.6	71.6	71.3	—	—	—	—

	Never Smoker	Former/Current Smoker			Non/Occasional Drinker		Regular Drinker	

Women								
Number of subjects	50,451	4,325			47,210		7,018	
Person-years	947,696.6	73,633.2			886,178.5		126,431.9	
Age, years, mean (SD)	52.1 (8.0)	50.0 (7.9)			52.3 (8.0)		49.1 (7.3)	
BMI, kg/m^2^, mean (SD)	23.4 (3.1)	23.1 (3.5)			23.5 (3.2)		22.9 (3.0)	
History of cholelithiasis, Yes, %	3.0	4.0			3.1		2.3	
History of diabetes mellitus, Yes, %	2.9	4.0			3.1		2.1	
History of chronic hepatitis or cirrhosis, Yes, %	0.9	1.8			—		—	
Japanese tea consumption, ≥1/day, %	75.2	69.7			74.8		74.5	
Current smoker, %	—	—			4.6		17.3	
Regular drinker, ≥1/week, %	11.0	34.8			—		—	

CI, confidence interval; HR, hazard ratio; IR, incidence ratio; SD, standard deviation.

The HRs and 95% CIs of the incidence of BTC and its subtypes (GBC, EHBDC, and AVC) and IHBDC associated with smoking in men are shown in Table 2. No clear association of smoking with BTC and its subtypes was observed. A significantly increased risk of IHBDC associated with smoking was observed (HR 2.25; 95% CI, 1.19–4.25 for current smokers with ≥30 pack-years compared with non-smokers, *P*-trend = 0.001).

**Table 2.** HRs and 95% CIs of the incidence of BTC and IHBDC according to smoking in men

	Cases	IR per 100,000	HR^a^	95% CI	

				Lower	Upper
IHBDC					
Never smoker	14	6.6	1.00		
Former smoker	12	6.0	0.82	0.38	1.79
Current smoker (<30 pack-years)	19	9.7	1.91	0.94	3.87
Current smoker (≥30 pack-years)	35	14.7	2.25	1.19	4.25
*P*-trend				0.001	

BTC					
Never smoker	67	31.4	1.00		
Former smoker	71	35.6	0.96	0.68	1.34
Current smoker (<30 pack-years)	36	18.3	0.82	0.54	1.24
Current smoker (≥30 pack-years)	72	30.3	0.94	0.67	1.32
*P*-trend				0.611	

Subtypes of BTC					
GBC					
Never smoker	18	8.4	1.00		
Former smoker	23	11.5	1.20	0.64	2.26
Current smoker (<30 pack-years)	10	5.1	0.89	0.41	1.97
Current smoker (≥30 pack-years)	19	8.0	0.99	0.51	1.91
*P*-trend				0.771	

EHBDC					
Never smoker	40	18.8	1.00		
Former smoker	39	19.6	0.83	0.53	1.31
Current smoker (<30 pack-years)	20	10.2	0.74	0.43	1.29
Current smoker (≥30 pack-years)	41	17.3	0.83	0.53	1.29
*P*-trend				0.400	

AVC					
Never smoker	5	2.3	1.00		
Former smoker	6	3.0	1.24	0.37	4.13
Current smoker (<30 pack-years)	5	2.5	1.50	0.42	5.29
Current smoker (≥30 pack-years)	10	4.2	2.09	0.70	6.27
*P*-trend				0.160	

AVC, ampulla of Vater cancer; CI, confidence interval; BTC, biliary tract cancer; EHBDC, extrahepatic bile duct cancer; GBC, gallbladder cancer; HR, hazard ratio; IHBDC, intrahepatic bile duct cancer; IR, incidence ratio.

^a^Adjusted for age, study area, body mass index, history of cholelithiasis, history of diabetes mellitus, history of chronic hepatitis or cirrhosis, Japanese tea consumption, and alcohol consumption.

The HRs and 95% CIs of the incidence of BTC and its subtypes and IHBDC associated with alcohol consumption in men are shown in Table 3. No clear association of alcohol consumption with BTC and its subtypes was observed. A non-significant trend of increased risk of IHBDC associated with increased alcohol consumption was observed (HR 1.96; 95% CI, 0.99–3.91 for regular drinkers with ≥300 g of ethanol consumption compared with non-drinkers, *P*-trend = 0.065).

**Table 3.** HRs and 95% CIs of the incidence of BTC and IHBDC according to alcohol consumption in men

	Cases	IR per 100,000	HR^a^	95% CI	

				Lower	Upper
IHBDC					
Non drinker	13	7.2	1.00		
Occasional drinker	7	9.1	1.57	0.62	3.98
Regular drinker (<300 g of ethanol)	32	8.8	1.51	0.78	2.89
Regular drinker (≥300 g of ethanol)	26	12.5	1.96	0.99	3.91
*P*-trend				0.065	

BTC					
Non drinker	61	33.7	1.00		
Occasional drinker	17	22.1	0.92	0.53	1.59
Regular drinker (<300 g of ethanol)	101	27.8	1.08	0.78	1.49
Regular drinker (≥300 g of ethanol)	57	27.4	1.04	0.71	1.52
*P*-trend				0.702	

Subtypes of BTC					
GBC					
Non drinker	19	10.5	1.00		
Occasional drinker	6	7.8	1.01	0.40	2.57
Regular drinker (<300 g of ethanol)	30	8.3	0.93	0.52	1.67
Regular drinker (≥300 g of ethanol)	12	5.8	0.71	0.34	1.51
*P*-trend				0.430	

EHBDC					
Non drinker	35	19.3	1.00		
Occasional drinker	7	9.1	0.72	0.32	1.63
Regular drinker (<300 g of ethanol)	53	14.6	1.05	0.68	1.63
Regular drinker (≥300 g of ethanol)	38	18.3	1.23	0.76	1.99
*P*-trend				0.362	

AVC					
Non drinker	7	3.9			
Occasional drinker	3	3.9	1.14	0.29	4.52
Regular drinker (<300 g of ethanol)	12	3.3	1.01	0.39	2.58
Regular drinker (≥300 g of ethanol)	4	1.9	0.56	0.16	1.99
*P*-trend				0.462	

AVC, ampulla of Vater cancer; CI, confidence interval; BTC, biliary tract cancer; EHBDC, extrahepatic bile duct cancer; GBC, gallbladder cancer; HR, hazard ratio; IHBDC, intrahepatic bile duct cancer; IR, incidence ratio.

^a^Adjusted for age, study area, body mass index, history of cholelithiasis, history of diabetes mellitus, history of chronic hepatitis or cirrhosis, Japanese tea consumption, and smoking.

The HRs and 95% CIs of the incidence of BTC and its subtypes and IHBDC associated with smoking stratified by drinking status (non-/occasional drinkers vs regular drinkers) in men are shown in Table 4. Current smokers with ≥30 pack-years had a non-significant trend of increased BTC risk compared with non-smokers among non-/occasional drinkers (HR 1.64; 95% CI, 0.94–2.86) but not among regular drinkers, and the interaction was marginally significant (*P*-interaction = 0.023). Current smokers had significantly increased risk of IHBDC among regular drinkers (HR 3.57; 95% CI, 1.39–9.22 for current smokers with <30 pack-years and HR 3.48; 95% CI, 1.41–8.61 for current smokers with ≥30 pack-years compared with non-smokers, *P*-trend = 0.001) but not among non-/occasional drinkers.

**Table 4.** HRs and 95% CIs of the incidence of BTC and IHBDC according to smoking stratified by drinking status in men

	Non/Occasional Drinker						Regular Drinker					
	
	Person-years	Cases	IR per 100,000	HR^a^	95% CI		Person-years	Cases	IR per 100,000	HR^b^	95% CI	
	
					Lower	Upper					Lower	Upper
IHBDC												
Never smoker	81,698.9	8	9.8	1.00			131,361.1	6	4.6	1.00		
Former smoker	59,790.5	2	3.3	0.30	0.06	1.44	139,454.3	10	7.2	1.47	0.53	4.07
Current smoker (<30 pack-years)	55,076.9	2	3.6	0.48	0.10	2.31	141,622.3	17	12.0	3.57	1.39	9.22
Current smoker (≥30 pack-years)	68,467.6	9	13.1	1.33	0.50	3.54	168,945.8	26	15.4	3.48	1.41	8.61
*P*-trend					0.461						0.001	
*P*-interaction											0.224	

BTC												
Never smoker	81,698.9	22	26.9	1.00			131,361.1	45	34.3	1.00		
Former smoker	59,790.5	23	38.5	1.11	0.61	2.02	139,454.3	48	34.4	0.87	0.58	1.32
Current smoker (<30 pack-years)	55,076.9	6	10.9	0.60	0.24	1.49	141,622.3	30	21.2	0.85	0.53	1.36
Current smoker (≥30 pack-years)	68,467.6	32	46.7	1.64	0.94	2.86	168,945.8	40	23.7	0.66	0.43	1.03
*P*-trend					0.117						0.068	
*P*-interaction											0.023	

Subtypes of BTC												
GBC												
Never smoker	81,698.9	6	7.3	1.00			131,361.1	12	9.1	1.00		
Former smoker	59,790.5	8	13.4	1.48	0.50	4.35	139,454.3	15	10.8	1.03	0.48	2.23
Current smoker (<30 pack-years)	55,076.9	0	0	0	—	—	141,622.3	10	7.1	1.06	0.45	2.52
Current smoker (≥30 pack-years)	68,467.6	12	17.5	2.41	0.88	6.58	168,945.8	7	4.1	0.44	0.17	1.14
*P*-trend					0.136						0.105	
*P*-interaction											0.040	

EHBDC												
Never smoker	81,698.9	14	17.1	1.00			131,361.1	26	19.8	1.00		
Former smoker	59,790.5	12	20.1	0.89	0.40	1.94	139,454.3	27	19.4	0.81	0.47	1.39
Current smoker (<30 pack-years)	55,076.9	5	9.1	0.75	0.27	2.11	141,622.3	15	10.6	0.71	0.37	1.36
Current smoker (≥30 pack-years)	68,467.6	15	21.9	1.15	0.55	2.41	168,945.8	26	15.4	0.68	0.39	1.19
*P*-trend					0.734						0.178	
*P*-interaction											0.347	

BTC, biliary tract cancer; CI, confidence interval; EHBDC, extrahepatic bile duct cancer; GBC, gallbladder cancer; HR, hazard ratio; IHBDC, intrahepatic bile duct cancer; IR, incidence ratio.

^a^Adjusted for age, study area, body mass index, history of cholelithiasis, history of diabetes mellitus, history of chronic hepatitis or cirrhosis, Japanese tea consumption, and alcohol consumption (non-drinker vs occasional drinker).

^b^Adjusted for age, study area, body mass index, history of cholelithiasis, history of diabetes mellitus, history of chronic hepatitis or cirrhosis, Japanese tea consumption, and alcohol consumption (<300 g/week of ethanol vs ≥300 g/week of ethanol).

The HRs and 95% CIs of the incidence of BTC and its subtypes and IHBDC associated with smoking and alcohol consumption in women are shown in Table 5. AVC was not analyzed because of the limited number of cases, and the analysis for smoking stratified by alcohol consumption was not conducted because female smokers and regular drinkers were too few to be analyzed. Clear association of each of smoking and alcohol consumption with these types of cancers was not observed in women.

**Table 5.** HRs and 95% CIs of the incidence of BTC and IHBDC according to smoking and alcohol consumption in women

	Cases	IR per 100,000	HR^a^	95% CI	

				Lower	Upper
Smoking					
IHBDC					
Never smoker	57	6.0	1.00		
Former/Current smoker	3	4.1	0.99	0.30	3.20

BTC					
Never smoker	213	22.5	1.00		
Former/Current smoker	14	19.0	1.16	0.67	2.01

Subtypes of BTC					
GBC					
Never smoker	111	11.7	1.00		
Former/Current smoker	8	10.9	1.30	0.62	2.70

EHBDC					
Never smoker	72	7.6	1.00		
Former/Current smoker	6	8.1	1.46	0.62	3.44

Alcohol					
IHBDC					
Non/Occasional drinker	56	6.3	1.00		
Regular drinker	2	1.6	0.43	0.10	1.79

BTC					
Non/Occasional drinker	206	23.2	1.00		
Regular drinker	20	15.8	1.02	0.63	1.63

Subtypes of BTC					
GBC					
Non/Occasional drinker	111	12.5	1.00		
Regular drinker	8	6.3	0.70	0.34	1.46

EHBDC					
Non/Occasional drinker	69	7.8	1.00		
Regular drinker	9	7.1	1.34	0.65	2.76

BTC, biliary tract cancer; CI, confidence interval; EHBDC, extrahepatic bile duct cancer; GBC, gallbladder cancer; HR, hazard ratio; IHBDC, intrahepatic bile duct cancer; IR, incidence ratio.

^a^Adjusted for age, study area, body mass index, history of cholelithiasis, history of diabetes mellitus, history of chronic hepatitis or cirrhosis, Japanese tea consumption, smoking (not applicable to the analysis for smoking) and alcohol consumption (not applicable to the analysis for alcohol).

## DISCUSSION

We investigated the association of smoking and alcohol consumption with the risks of BTC and its subtypes and IHBDC incidence in men and women in a large-scale population-based prospective cohort study in Japan. The results showed a different effect of smoking and alcohol consumption between IHBDC and BTC in men; an increased risk of IHBDC was significantly associated with smoking, especially among regular drinkers, and tended to be associated with alcohol consumption; an increased risk of BTC associated with smoking and alcohol consumption was not observed in all participants, although a non-significant trend of increased risk of BTC associated with smoking was observed only in non-/occasional drinkers. The association of smoking and alcohol consumption in women was unclear because the proportion of current smokers and regular drinker was very limited.

The strengths of the present study are its large sample size and prospective design. Associations between BTC and alcohol consumption/smoking have been investigated in a previous study using data from the same cohort.^4^ The follow-up period was extended by around 8 years since then, and the number of cases has increased, which enabled the present study to perform more detailed analysis, including stratification by drinking status, analysis for each of the BTC subtypes, and the evaluation of dose response with smoking intensity or alcohol volume. To our knowledge, the sample size of the present study is the largest among the epidemiological studies that have investigated the association between BTC and smoking or alcohol consumption.

The present study showed a null effect of smoking on BTC, while increased risk of IHBDC was observed in men. It is interesting that different effects of smoking were observed between IHBDC and BTC when stratified by drinking status; a nonsignificant trend of increased risk of BTC in non-/occasional drinkers and no association in regular drinkers, while we found increased risk of IHBDC in regular drinkers and no association in no/occasional drinkers. This finding may suggest that, although smoking has the effect of increasing BTC risk, alcohol may neutralize the smoking effect in BTC. With respect to the interaction between alcohol and smoking, alcohol may potentially have the dual effect of enhancing the smoking effect by inducing enzymes (eg, CYP2E1) to metabolize procarcinogens into carcinogens, and of neutralizing the smoking effect by inducing enzyme systems responsible for detoxification of those produced carcinogens.^24^ Our findings suggest that induction of enzymes related to carcinogen metabolism by alcohol may differ between organs (liver and biliary tract). Further investigation in other cohort or case-control studies is needed to confirm our results.

Previous studies of the association between smoking and BTC were inconsistent regardless of total BTC,^4^^,^^22^^,^^23^^,^^30^ GBC,^16^ or EHBDC.^15^ Most of them were retrospective in design and did not stratify by drinking status. There was only one study that investigated the effects of smoking stratified by drinking status, in which an increased risk associated with smoking was observed only among non-drinkers,^23^ which is consistent with the present study. For IHBDC, an increased risk was observed in three out of eight previous studies, and a meta-analysis showed a non-significant trend of increased risk (RR 1.35; 95% CI, 0.95–1.82), although dose dependency was not investigated.^7^ The present study is the first prospective assessment of the dose-response relationship, and the result suggests that heavier smokers, especially among regular drinkers, may have a higher risk.

The present study showed no clear association of alcohol consumption with BTC, while a non-significant trend of increased risk of IHBDC was observed in men. Alcohol consumption was not associated with IHBDC risk among never smokers when stratified by smoking status (HR 0.61; 95% CI, 0.12–3.09 for regular drinkers with ≥300 g of ethanol consumption), suggesting that the observed increased risk of IHBDC may be due to residual confounding by smoking or interaction between smoking and alcohol consumption. For IHBDC, 10 case-control studies have investigated the association with alcohol consumption, and an increased risk was observed in six studies.^13^

The present study is the first prospective study of the association of smoking and drinking with IHBDC, and showed the similar result of increased IHBDC risk to many of the previous studies although it was not statistically significant due to the limited sample size. For BTC, a null effect of alcohol consumption has been consistently observed in the previous epidemiological studies,^4^^,^^15^^,^^20^^–^^23^ with the exception of one prospective study in Japan, in which increased risk of GBC was observed.^31^ The present study is the largest study for BTC and supported the result of null effect. It is not clear why a different effect was observed between two prospective studies in Japan, but it may be attributable to differences in the characteristics of the study population (eg, the proportion of regular drinkers and the amount of ethanol consumption were less in the present study).

The present study has several limitations. First, despite the large-scale design and sufficient sample size of total BTCs, the sample sizes of IHBDC was limited. Second, there could be some misclassification in the exposure category because the data obtained at only a single point using a baseline survey for exposure classification, and relevant changes in lifestyle during the follow-up were not reflected in the classification. Third, there could be some effect of unmeasured variables and residual confounding, although the statistical model was adjusted for as many variables as possible. For example, this study does not have information about relevant medical history, such as anoumalous arrangement of pancreaticobiliary duct and primary biliary cholangitis. In addition, we did not use information on hepatitis virus infection in this study because it is available for only one fifth of subjects. In the future study, this information should be collected and the multivariate model should be adjusted for these variables. Fourth, in the present cohort, the number of female current smokers and regular drinkers were exceedingly small, resulting in an unclear effect of smoking and alcohol consumption in women.

In conclusion, in a population-based, prospective cohort study in Japan, smoking was significantly associated with an increased risk of IHBDC in men, especially among regular drinkers. On the other hand, smoking and alcohol consumption were not associated with BTC risk.

## APPENDIX

Members of the Japan Public Health Center-based Prospective Study (JPHC Study, principal investigator: S. Tsugane) Group are: S. Tsugane, N. Sawada, M. Iwasaki, S. Sasazuki, T. Yamaji, T. Shimazu, and T. Hanaoka, National Cancer Center, Tokyo; J. Ogata, S. Baba, T. Mannami, A. Okayama, and Y. Kokubo, National Cerebral and Cardiovascular Center, Osaka; K. Miyakawa, F. Saito, A. Koizumi, Y. Sano, I. Hashimoto, T. Ikuta, Y. Tanaba, H. Sato, Y. Roppongi, T. Takashima, and H. Suzuki, Iwate Prefectural Ninohe Public Health Center, Iwate; Y. Miyajima, N. Suzuki, S. Nagasawa, Y. Furusugi, N. Nagai, Y. Ito, S. Komatsu, and T. Minamizono, Akita Prefectural Yokote Public Health Center, Akita; H. Sanada, Y. Hatayama, F. Kobayashi, H. Uchino, Y. Shirai, T. Kondo, R. Sasaki, Y. Watanabe, Y. Miyagawa, Y. Kobayashi, M. Machida, K. Kobayashi, and M. Tsukada, Nagano Prefectural Saku Public Health Center, Nagano; Y. Kishimoto, E. Takara, T. Fukuyama, M. Kinjo, M. Irei, and H. Sakiyama, Okinawa Prefectural Chubu Public Health Center, Okinawa; K. Imoto, H. Yazawa, T. Seo, A. Seiko, F. Ito, F. Shoji, and R. Saito, Katsushika Public Health Center, Tokyo; A. Murata, K. Minato, K. Motegi, T. Fujieda, and S. Yamato, Ibaraki Prefectural Mito Public Health Center, Ibaraki; K. Matsui, T. Abe, M. Katagiri, M. Suzuki, and K. Matsui, Niigata Prefectural Kashiwazaki and Nagaoka Public Health Center, Niigata; M. Doi, A. Terao, Y. Ishikawa, and T. Tagami, Kochi Prefectural Chuo-higashi Public Health Center, Kochi; H. Sueta, H. Doi, M. Urata, N. Okamoto, F. Ide, H. Goto, and R Fujita, Nagasaki Prefectural Kamigoto Public Health Center, Nagasaki; H. Sakiyama, N. Onga, H. Takaesu, M. Uehara, T. Nakasone, and M. Yamakawa, Okinawa Prefectural Miyako Public Health Center, Okinawa; F. Horii, I. Asano, H. Yamaguchi, K. Aoki, S. Maruyama, M. Ichii, and M. Takano, Osaka Prefectural Suita Public Health Center, Osaka; Y. Tsubono, Tohoku University, Miyagi; K. Suzuki, Research Institute for Brain and Blood Vessels Akita, Akita; Y. Honda, K. Yamagishi, S. Sakurai, and N. Tsuchiya, University of Tsukuba, Ibaraki; M. Kabuto, National Institute for Environmental Studies, Ibaraki; M. Yamaguchi, Y. Matsumura, S. Sasaki, and S. Watanabe, National Institute of Health and Nutrition, Tokyo; M. Akabane, Tokyo University of Agriculture, Tokyo; T. Kadowaki and M. Inoue, The University of Tokyo, Tokyo; M. Noda and T. Mizoue, National Center for Global Health and Medicine, Tokyo; Y. Kawaguchi, Tokyo Medical and Dental University, Tokyo; Y. Takashima and Y. Yoshida, Kyorin University, Tokyo; K. Nakamura and R. Takachi, Niigata University, Niigata; J. Ishihara, Sagami Women’s University, Kanagawa; S. Matsushima and S. Natsukawa, Saku General Hospital, Nagano; H. Shimizu, Sakihae Institute, Gifu; H. Sugimura, Hamamatsu University School of Medicine, Shizuoka; S. Tominaga, Aichi Cancer Center, Aichi; N. Hamajima, Nagoya University, Aichi; H. Iso and T. Sobue, Osaka University, Osaka; M. Iida, W. Ajiki, and A. Ioka, Osaka Medical Center for Cancer and Cardiovascular Disease, Osaka; S. Sato, Chiba Prefectural Institute of Public Health, Chiba; E. Maruyama, Kobe University, Hyogo; M. Konishi, K. Okada, and I. Saito, Ehime University, Ehime; N. Yasuda, Kochi University, Kochi; S. Kono, Kyushu University, Fukuoka; S. Akiba, Kagoshima University, Kagoshima; T. Isobe, Keio University; Y. Sato, Tokyo Gakugei University.
